# miRNA Profiles as a Predictor of Chemoresponsiveness in Wilms’ Tumor Blastema

**DOI:** 10.1371/journal.pone.0053417

**Published:** 2013-01-07

**Authors:** Jenny A. Watson, Kenneth Bryan, Richard Williams, Sergey Popov, Gordan Vujanic, Aurore Coulomb, Liliane Boccon-Gibod, Norbert Graf, Kathy Pritchard-Jones, Maureen O’Sullivan

**Affiliations:** 1 The National Children’s Research Centre, Our Lady’s Children’s Hospital, Crumlin, Dublin, Ireland; 2 Cancer Genetics, Royal College of Surgeons in Ireland, Dublin, Ireland; 3 UCL Institute of Child Health, London, United Kingdom; 4 The Institute of Cancer Research, Surrey, United Kingdom; 5 School of Medicine, Cardiff University, Cardiff, United Kingdom; 6 Hôpital Armand-Trousseau, Université Pierre et Marie Curie-Paris, Paris, France; 7 Department of Pediatric Oncology and Hematology, University of Saarland, Homburg, Germany; 8 Department of Pathology, Our Lady’s Children’s Hospital, Crumlin, Dublin, Ireland; University of Florida, United States of America

## Abstract

The current SIOP treatment protocol for Wilms’ tumor involves pre-operative chemotherapy followed by nephrectomy. Not all patients benefit equally from such chemotherapy. The aim of this study was to generate a miRNA profile of chemo resistant blastemal cells in high risk Wilms’ tumors which might serve as predictive markers of therapeutic response at the pre-treatment biopsy stage. We have shown here that unsupervised hierarchical clustering of genome-wide miRNA expression profiles can clearly separate intermediate risk tumors from high risk tumors. A total of 29 miRNAs were significantly differentially expressed between post-treatment intermediate risk and high risk groups, including miRNAs that have been previously linked to chemo resistance in other cancer types. Furthermore, 7 of these 29 miRNAs were already at the pre-treatment biopsy stage differentially expressed between cases ultimately deemed intermediate risk compared to high risk. These miRNA alterations include down-regulation in high risk cases of miR-193a.5p, miR-27a and the up-regulation of miR-483.5p, miR-628.5p, miR-590.5p, miR-302a and miR-367. The demonstration of such miRNA markers at the pre-treatment biopsy stage could permit stratification of patients to more tailored treatment regimens.

## Introduction

Wilms’ Tumor (nephroblastoma), the commonest pediatric renal tumor, arises predominantly in children under 5 years old, and is thought to develop from embryonic kidney cells [1], [2]. Of all the embryonal tumors, Wilms’ represents the best example of organogenesis ”gone awry”, as it contains varying amounts of the triphasic elements of nephrogenesis including epithelial, stromal and blastemal cell types, which are recognisable by light microscopy.

Overall, outcomes for Wilms’ tumor are excellent and a current key aim is the minimisation of therapy in these very young patients to avoid long-term sequelae [3]. The current SIOP (Societe Internationale d’Oncologie Paediatrique) protocol for the treatment of Wilms’ Tumor in patients over the age of 6 months includes neoadjuvant chemotherapy followed by nephrectomy [4]. The rationale is to down-stage tumors pre-operatively in order to facilitate safer nephrectomy [5], [6]. Post-nephrectomy, histological evaluation permits staging and risk-based stratification. According to the CCLG [Children’s Cancer and Leukaemia Group] protocol, up-front diagnostic biopsy of the tumor is additionally performed; thus therapy-naïve tissue is evaluable in such cases. There are currently no means at biopsy stage of identifying which cases will be ‘blastemal’ and so deemed high-risk at nephrectomy. Along with morphologically identified diffusely anaplastic cases [known to be associated with TP53 mutation], blastemal Wilms’ represent the chemo-resistant cohort requiring more intensive adjuvant chemotherapy. Studies that consider the molecular mechanisms of chemo-resistance in Wilms’ tumor have focused almost exclusively on gene expression profiling with no evidence of signatures that might predict chemotherapeutic response [7]. Evidence is emerging for the role of miRNAs in both renal development [8] and Wilms’ tumor progression [9]. Given the lack of clear genomic/genetic pointers, our aim was to identify markers of chemo resistance in Wilms’ tumor by miRNA profiling.

## Materials and Methods

### Patient Samples

In this study, the term chemo responsiveness is based on histological findings such that intermediate risk cases are considered to have shown better response, while high risk cases showed relative resistance. The patient cohort (Table 1) initially included 18 pre-treatment biopsy specimens and 27 post-treatment nephrectomy specimens taken from patients who had undergone neoadjuvant chemotherapy. Of these post-treatment specimens, 13 represented intermediate risk cases [Post-IR-NBl], where a response to chemotherapy was evidenced histologically by the presence of cellular maturation whereas persistent blastema was of limited proportion. The remaining 14 specimens were deemed high risk [Post-HR] or non-chemo responsive, due to blastemal histology. The pre-treatment group comprised 4 tumor biopsy samples from patients who were ultimately deemed high risk [Pre-HR] and 14 which were ultimately intermediate risk [Pre-IR]. Full face unstained sections of formalin-fixed, paraffin-embedded tissue were cut, and following the review of corresponding haematoxylin and eosin-stained sections, blastemal cells were selectively isolated by macro-dissection from all pre-treatment biopsies [Pre-IR and Pre-HR] and the 14 post-treatment high risk specimens [Post-HR]. For the 13 post-treatment intermediate risk specimens, the epithelial and/or stromal components were isolated for analysis [Post-IR-NBl]. Subsequently, an additional group of 12 post-treatment intermediate risk cases were added to the cohort, from which blastemal tissue was selectively isolated [Post-IR-Bl].

**Table 1:** Sample Cohort.

Group name	Short name	Sample source	Cell type	Treatment administration	Sample number
Pre-treatment High-risk	Pre-HR	Biopsy	Blastemal	Pre-treatment	4
Pre-treatment Intermediate-risk	Pre-IR	Biopsy	Blastemal	Pre-treatment	14
Post-treatment High-risk	Post-HR	Nephrectomy	Blastemal	Post-treatment	14
Post-treatment Intermediate-risk	Post-IR-NBl	Nephrectomy	Epithelial/Stromal	Post-treatment	13
Post-treatment Intermediate-risk	Post-IR-Bl	Nephrectomy	Blastemal	Post-treatment	12

### miRNA Profiling Using TaqMan Low Density Arrays

Total RNA was extracted according to the manufacturer’s instructions using RecoverAll Total Nucleic Acid Isolation Kit (Ambion) which recovers the small RNA fraction and allows for the downstream analysis of miRNAs. RNA was reverse transcribed using the miRNA reverse transcription kit (Applied Biosystems). Megaplex primer pool A used in this reaction corresponds to TaqMan Low Density Array (TLDA) A (Applied Biosystems), and allows the simultaneous reverse transcription of 377 miRNAs, 3 endogenous controls and 1 miRNA assay unrelated to mammalian species on TLDA A. Alternatively, for the individual analysis of miRNAs using Taqman miRNA assays, target-specific stem-loop RT primers were used in combination with the miRNA reverse transcription kit. Megaplex RT product was pre-amplified using TaqMan PreAmp Master Mix (2×) and PreAmp Primer Pool A (Applied Biosystems). The PreAmp primer pool contains forward primers specific for the miRNAs on TLDA A and a universal reverse primer (Applied Biosystems). Each cDNA sample was analyzed on TLDA A using the 7900HT RT-qPCR system (Applied Biosystems).

### Statistical and Bioinformatic Analysis

Ct values and Relative quantities were calculated using RQ Manager 1.2.1 with the application of automatic baseline settings and a threshold of 0.2. Prior to analysis samples were first standardized (mean centred) and the normalized relative expression (NRQ) values of miRNA were calculated with reference to the Ct _max_ (maximum Ct value of a miRNA across samples) using the equation NRQ  = 2(Ct_max_−Ct). MiRNAs that were expressed in at least 10 samples were retained for analysis.

The combined hierarchical clustering was carried out using Spearman’s rank correlation as an object similarity metric and complete linkage was implemented to ascertain cluster similarity using the *hclust* function. The associated heatmap analysis was implemented using the *heatmap.plus* function. Colors were generated in a standardized manner using the rank of miRNA expression values across samples.

The non-parametric Wilcoxon’s rank sum was used to measure the significance of association of miRNA expression to the various selected sample classes. P-values were corrected for multiple comparisons using the Bonferroni method. Potential targeting of differentially expressed miRNA and diametrically, differentially expressed mRNA was examined by assessing the number of predicted targets present. The significance of this value was calculated by comparing this with that expected by chance using Chi-squared statistic. This analysis was carried out using an algorithm implemented Java v1.5 and the TargetScan version 5.1 database.

## Results

In this study, the intermediate risk cases were defined, as patients whose tumors were histologically regressive, epithelial, stromal or mixed, post-chemotherapy. Of these, 69% were of mixed histology, 15% stromal, 8% epithelial and 8% regressive. The high risk group comprised blastemal cases only. Anaplastic cases were deliberately excluded from this analysis, on the basis that these tumors are driven, at least in part, by TP53 mutation and are also readily recognisable microscopically. This pathological stratification parallels the clinical outcomes. Within our high risk blastemal group, 28.6% of patients relapsed and a 78.6% 2 year survival is evident, whereas within the intermediate risk group, there is a 100% 2 year survival and only 15.4% of patients relapsed. The average ages of patients within high and intermediate risk groups are 46 months and 45 months respectively.

**Figure 1:**
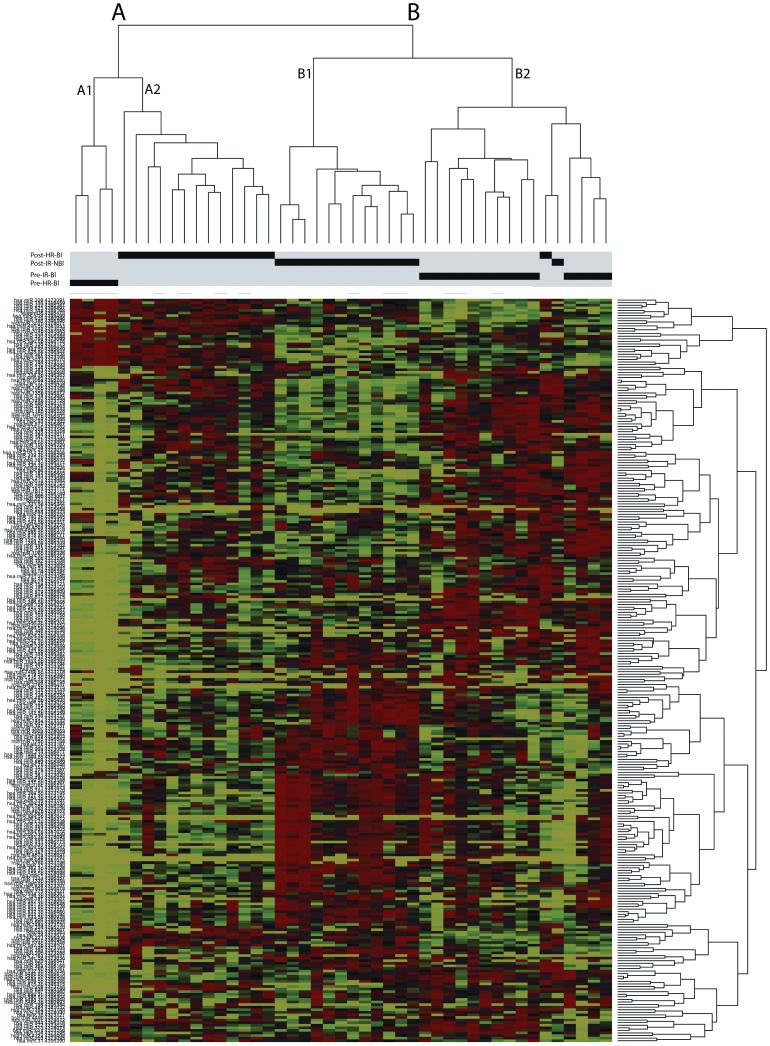
Unsupervised hierarchical clustering analysis. Of the 295 miRNAs, clustering analysis separates tumors into two clusters that clearly segregate intermediate risk from high risk cases. Cluster A includes 13 post-treatment and 4 pre-treatment high risk cases, and cluster B includes 13 post-treatment and 14 pre-treatment intermediate risk cases. 4 sub-clusters further discriminate between pre- and post- treatment high risk (A1 and A2) and pre- and post- treatment intermediate risk groups (B2 and B1).

In order to assess the miRNA profiles of intermediate and high risk groups, genome-wide miRNA expression analysis was performed using TaqMan Low Density Array A, which includes 377 miRNAs and of these, 295 miRNAs were retained for analysis. Unsupervised hierarchical clustering performed on these 295 miRNAs separates tumors into two clusters that clearly discriminate high risk [A] from intermediate risk [B] cases. Sub-clusters further discriminate between 4 pre- and 13 post-treatment high risk groups (A1 and A2) and again between 14 pre- and 13 post-treatment intermediate risk groups (B2 and B1). Only one post-treatment high risk tumor was mis-classified by this analysis (Figure 1).

Following these initial observations, further unsupervised clustering analysis was also performed. This analysis includes an additional group of post-treatment intermediate risk cases, from which the residual blastemal component had been isolated [Post-IR-Bl], and results in the disruption of the distinct clustering patterns observed between intermediate and high-risk groups in Figure 1. Based on this analysis of miRNA expression profiles, blastema from post-treatment intermediate risk cases [Post-IR-Bl], appears most similar to blastema originating from pre-treatment high risk blastemal cases [Pre-HR]. This is illustrated as supporting information in Figure S1.

We assessed the miRNA expression differences between residual blastema isolated from post-treatment intermediate risk cases [Post-IR-Bl] and post-treatment high risk blastema [Post-HR-Bl]. This analysis identified 10 miRNAs (miR-551b, miR-106b, miR-24, miR-542.3p, miR-331.3p, miR-223, miR-518b, miR-25, miR-30b, miR-523) with significantly higher expression in high risk cases (Table 2). The assumption here is that such miRNAs somehow confer survival/proliferative advantage on blastemal cells, allowing more thereof to survive chemotherapy in the high-risk group.

**Table 2:** miRNAs significantly up-regulated within the blastemal component of post-treatment high risk cases [Post-HR] when compared to post-treatment intermediate risk cases [Post-IR-Bl].

miRNA ID	Wilcoxon Rank [Corrected]
hsa.miR.551b	1.22E-04
hsa.miR.106b	2.75E-03
hsa.miR.24	4.09E-03
hsa.miR.542.3p	5.93E-03
hsa.miR.331.3p	8.49E-03
hsa.miR.223	8.49E-03
hsa.miR.518b	9.85E-03
hsa.miR.25	1.66E-02
hsa.miR.30b	2.27E-02
hsa.miR.523	4.13E-02

The principal aim of this study was to identify miRNAs that are predictive of chemotherapeutic response at the biopsy stage, and which might then be used to stratify patients *prior* to the administration of pre-nephrectomy chemotherapy. In order to address this, we compared miRNA profiles of the non-blastemal elements of post-treatment intermediate risk cases [Post-IR-NBl], with post-treatment high risk tumor blastema [Post-HR]; essentially comparing cells that have shown response with those that have not. A total of 29 miRNAs differentially expressed between non-blastemal elements of intermediate risk cases (Post-IR-NBl) and high risk blastemal cases were identified (excluding major and minor forms of the same miRNA), with 15 miRNAs down-regulated and 14 miRNAs up-regulated in post-treatment high risk [Post-HR] cases (p = ≤0.05) (Tables 3). We investigated for representation of these miRNAs at the biopsy stage, through comparison of pre-treatment biopsies originating from intermediate risk cases [Pre-IR], to pre-treatment biopsies from high risk cases [Pre-HR]. Interestingly, 7 miRNAs from the post-treatment analysis were also differentially expressed at the biopsy stage (Table 4). Although the pre-treatment analysis did not yield any significant p-values, perhaps due to the small sample size of the pre-treatment high risk group, a total of 54 miRNAs approached significance (p = 0.19).

**Table 3:** miRNAs significantly differentially expressed in post-treatment high-risk cases [Post-HR] when compared to post-treatment intermediate-risk cases [Post-IR-NBl].

miRNA ID	Wilcoxon Rank [Corrected]
miRNAs significantly down-regulated in post-treatment high-risk cases	
hsa.miR.193a.5p	5.88E-05
hsa.miR.369.3p	5.88E-05
hsa.miR.369.5p	2.03E-02
hsa.miR.455.3p	8.00E-03
hsa.miR.455.5p	3.50E-02
hsa.miR.22	8.00E-03
hsa.miR.27a	1.10E-02
hsa.miR.654.3p	1.25E-02
hsa.miR.99a	1.49E-02
hsa.miR.574.3p	2.00E-02
hsa.miR.100	2.66E-02
hsa.miR.199b.5p	2.66E-02
hsa.miR.154	3.43E-02
hsa.miR.145	3.50E-02
hsa.miR.502.3p	4.58E-02
hsa.miR.494	4.58E-02
hsa.miR.199a.5p	4.58E-02
**miRNAs significantly up-regulated in post-treatment high-risk cases**	
hsa.miR.483.5p	1.18E-04
hsa.miR.93	1.32E-03
hsa.miR.520e	2.85E-03
hsa.miR.628.5p	3.31E-03
hsa.miR.590.5p	4.09E-03
hsa.miR.491.5p	4.09E-03
hsa.miR.302a	5.74E-03
hsa.miR.17	1.10E-02
hsa.miR.106a	1.10E-02
hsa.miR.125a.5p	1.49E-02
hsa.miR.19a	2.00E-02
hsa.miR.106b	2.66E-02
hsa.miR.20a	4.58E-02
hsa.miR.367	4.58E-02

**Table 4:** miRNAs significantly differentially expressed in both pre-treatment high risk group [Pre-HR] compared to pre-treatment intermediate risk group [Pre-IR], and also in the post-treatment high-risk group [Post-HR] compared to the post-treatment intermediate risk group [Post-IR-NBl].

		PRE-TREATMENT	POST-TREATMENT		
miRNA ID	Expression in high risk cases compared to intermediate risk	Wilcoxon Rank [Corrected]	Average miRNA expression in intermediaterisk cases	Average miRNA expression in highrisk cases	Wilcoxon Rank [Corrected]
hsa.miR.193a.5p	Down regulated	5.88E-05	650.62	83.92	1.93E-01
hsa.miR.27a		1.10E-02	142.39	70.8	1.93E-01
hsa.miR.483.5p	Up regulated	1.18E-04	54.08	792.19	1.93E-01
hsa.miR.628.5p		3.31E-03	4289.53	10867785.86	1.93E-01
hsa.miR.590.5p		4.09E-03	2.529	15.305	1.93E-01
hsa.miR.302a		5.74E-03	198.01	3763.27	1.93E-01
hsa.miR.367		4.58E-02	6.64	70.20	1.93E-01

In order to focus on potential markers of chemo resistance in Wilms’ tumor we considered further the 7 miRNAs that were found in this study to be significantly altered between intermediate risk and high risk groups post-treatment, and also already differentially expressed in the pre-treatment analyses. Our assumption is that these miRNAs may be particularly important indicators of the tumor’s potential to respond to pre-operative chemotherapy. These miRNA alterations included the down-regulation of miR-193a.5p and miR-27a, and the up-regulation of miR-483.5p, miR-628.5p, miR-590.5p, miR-302a and miR-367 in high risk cases (Table 4). Although miR-20a expression was altered in both pre- and post-treatment analyses the directional changes were not consistent and therefore this miRNA was not considered further.

## Discussion

According to the SIOP protocol, standard management of renal tumors is pre-operative chemotherapy followed by nephrectomy. Although in most cases of Wilms’ tumor such therapy induces a considerable degree of tumor shrinkage with evidence of necrosis and cellular differentiation, a proportion of cases show lesser volume reduction and retain an abundance of blastemal tissue post-chemotherapy and these are stratified as high-risk blastemal cases and have a poorer prognosis [4].

To date, there is a very limited understanding of the molecular effects of therapy on Wilms’ tumor and the factors that produce chemo resistance in this tumor. This is partly due to challenges in the acquisition of paired pre-and post-treatment cases, as the National Wilms tumor study group (NWTS) in North America currently recommends primary surgical resection of Wilms’ tumors and even within the SIOP protocol, only a few countries offer biopsy-based diagnosis prior to treatment.

Previous studies have focused on the assessment of histological features in pre- versus post-treatment Wilms’ tumor cases [10], [11]. Barroca (2010), found that the cytologic features of pre-treatment fine needle biopsies did not have any bearing on the type and extent of post-treatment regressive changes, however, all predominantly blastemal (high risk) post-treatment tumors corresponded to pre-treatment cases with considerable blastemal components and little necrosis or apoptosis. In order to explain this phenomenon, Barroca suggested that two types of blastema may occur in Wilms’ tumor and that each of these types exhibits different chemotherapeutic sensitivities, proliferative properties and abilities to undergo apoptosis or necrosis [11]. Although no molecular analysis has been conducted to date that confirms this theory.

In this study we performed a direct comparison of miRNA profiles between post-treatment blastema, isolated from intermediate risk cases and the predominant blastemal component in high risk cases and identified 10 miRNAs to be significantly up-regulated in the high risk cases (Table 2). Of particular interest are the up-regulation of miRNA 106b and miR-25 in the high risk blastema. These miRNAs are members of the miR-106b∼25 cluster located within intron 13 of the *Mcm7* gene that has been found to directly target a number of important tumor suppressor pathways and may therefore be involved in the promotion of cellular proliferation in high risk blastemal regions [12]. For instance, miR-106b directly targets Retinoblastoma [RB], tumor suppressor gene PTEN [13], as well as cyclin-dependent kinase inhibitor p21/CDKN1A, [14] and pro-apoptotic Bcl-2 family member BCL2L11 [Bim] [12]. The suppression of even one of these pathways by the over-expression of miR-106b identified here, has the potential to lead to a highly proliferative blastema with reduced apoptosis and could thereby contribute to the persistent and voluminous nature of this blastemal component in high risk cases.

A number of recent publications suggest a significant role for miRNAs in the molecular response to chemotherapy and a selection of miRNAs has now been associated with chemo resistant phenotypes in a range of cancer types [15], [16]. Varying levels of chemo sensitivity may be inferred by the influence of miRNA up- or down-regulation on multiple mRNA targets and subsequent protein expression. This may impact the cellular response to anticancer agents by the dysregulation of survival pathways, apoptotic capabilities, specific drug targets, DNA repair systems or drug transport/metabolism [17], [18]. However, it has also been shown that the role of miRNAs in chemo resistance is complex and often miRNAs are seen to have the opposite effects towards similar anticancer agents in different cancer types [19]. A number of differentially expressed miRNAs identified in this study has been previously associated with chemo resistance. For example, miR-15b, which is down-regulated in pre-treatment high risk cases here, has previously been shown to play a role in the development of multi-drug resistance in gastric cancer cells through up-regulation of the Bcl2 protein and consequent modulation of apoptotic pathways [20]. Alternatively, up-regulation of miR-222 is also known to confer Tamoxifen drug-resistance in breast cancer cells through the negative regulation of ERα and cell cycle inhibitor p27Kip1 [21], [22].

In this study, in order to establish a potential miRNA marker that may be predictive of chemotherapy response at biopsy stage, we selected miRNAs where expression was altered between intermediate and high risk cases not only *after*, but also already *before* chemotherapy. The assumption here is that any marker that might indicate a resistant tumor type prior to treatment would remain detectable in high risk cases, as it is precisely the tumor cells expressing this marker that would be chemo resistant.

Here we have highlighted a signature of 7 differentially expressed miRNAs within pre-treatment diagnostic biopsy specimens that may be predictive of chemo resistance in Wilms’ tumor. These include the down-regulation of miR-193a.5p and miR-27a, and the up-regulation of miR-483.5p, miR-628.5p, miR-590.5p, miR-302a and miR-367. Of particular interest are the protein targets of miR-27a, miR-302 and miR-483.5p. The down-regulation of miR-27a has been linked to p-glycoprotein expression and has previously been reported to correlate with an up-regulation of this protein and to contribute to chemo resistance and relapse in leukemia patients [23]. The principal role of p-glycoprotein is to mediate the elimination of toxic substances from the cell. It is normally expressed in kidney cells and its expression has been shown to be enhanced by Vincristine and Dactinomycin chemotherapy in Wilms’ tumor patients [24].

It is plausible that the over-expression of miR-302 could promote an embryonic stem cell-like cell cycle profile through the suppression of cyclin D1 and Cdk4 [25]. This is particularly applicable here as Wilms’ tumor likely arises from multipotent embryonic kidney precursor cells and the expression of residual multipotent properties may remain evident within regions of persistent blastema that are a morphological feature of chemo-resistant histology. In addition, miR-367 is part of the miR-302 cluster, and the miR-302/367 promoter is known to be transcriptionally regulated by embryonic stem cell-specific transcription factors [26]. These miRNAs are expressed at high levels in post-treatment high risk cases in this study and may therefore confer multipotent properties on these blastemal cells [27].

miR-483.5p upregulated in our high risk cases, is located within intron 2 of the *IGF2* gene on chromosome 11p15 [28] and is thought to be co-expressed with its *IGF2* host gene [29]. As the expression of *IGF2* has a particularly significant role in Wilms’ tumorigenesis and is often increased due to associated loss of imprinting (LOI) events [30], the expression of this miRNA may therefore also be frequently affected in tandem with IGF2 in Wilms’ tumor, although this has not been studied to-date. miR-483-5p has been shown to target the *suppressor of cytokine signalling 3 (SOCS3)* [31]. This results in the decreased expression of the SOCS3 protein leading to sustained signaling of the *Interleukin-6 (IL-6)*/*Signal transducers and activators of transcription 3 (STAT3)* cytokine pathways which stimulates growth and proliferation and is potentially anti-apoptotic [32].

It is possible that one of the differentially expressed miRNAs identified in this study may alone be the key component in determining response to chemotherapy in Wilms’ tumor patients. But, perhaps these miRNAs in fact work in tandem in regulating the complex pathways of chemotherapeutic response and may need to be considered as a miRNA signature in future studies. In order to validate the miRNAs highlighted here, a prospective study would need to be undertaken wherein the expression of these miRNAs is assessed in patient biopsy samples. The suggestion here is that miRNA markers identifiable in pre-treatment biopsy specimens could allow for early prediction of potential drug resistance and may be an important step towards customizing current regimens in non-responsive Wilms’ tumor patients.

## Supporting Information

Figure S1 **Unsupervised hierarchical clustering of miRNAs including additional intermediate risk group with selected blastemal components.**
(TIF)
