# Supplementary figures and images for: miRNA Profiles as a Predictor of Chemoresponsiveness in Wilms’ Tumor Blastema
Source: PLoS One. 2013 Jan 7;8(1):e53417. doi: 10.1371/journal.pone.0053417 (PMC3538586; doi:10.1371/journal.pone.0053417)

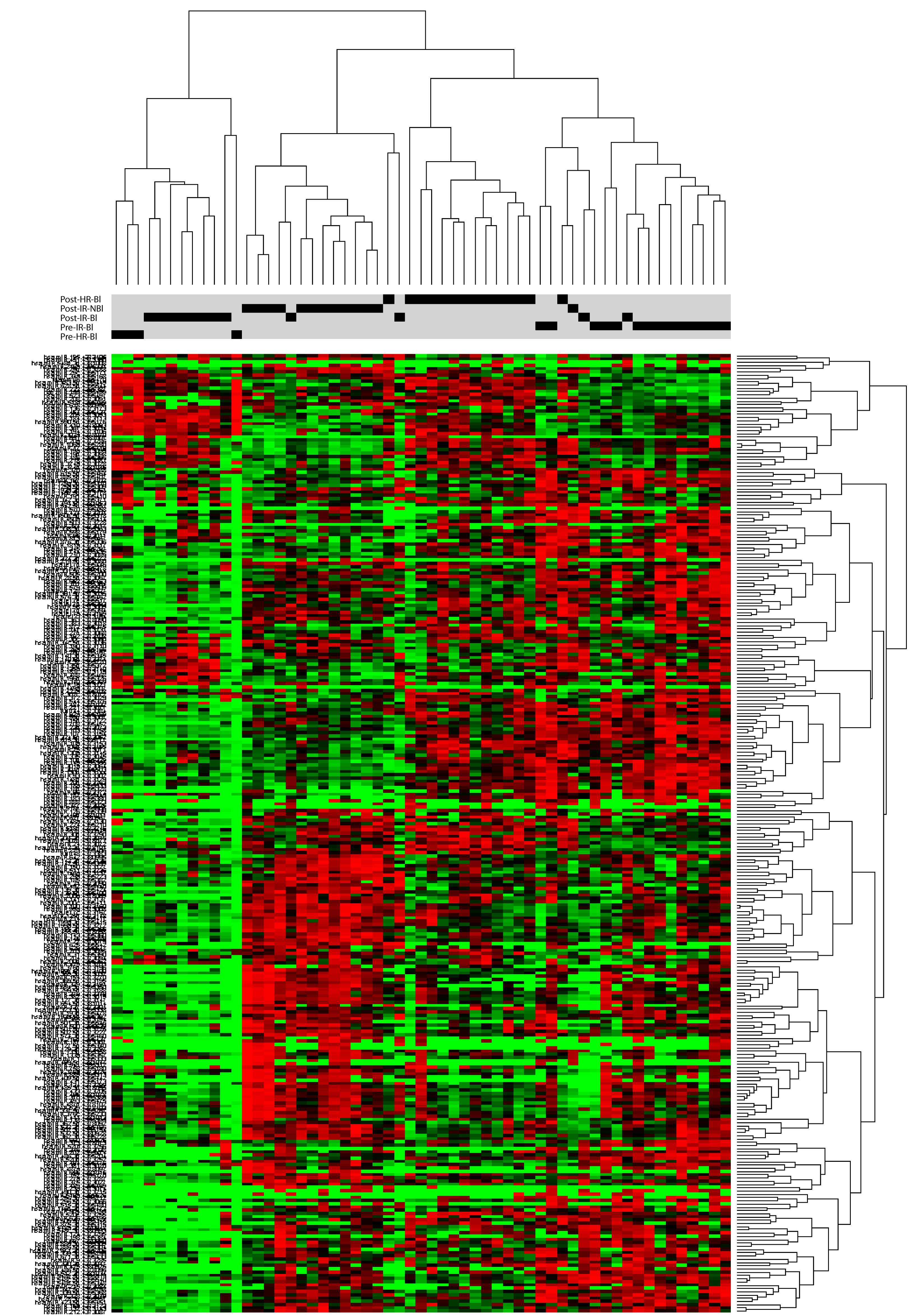

Supplement: Figure S1 — Unsupervised hierarchical clustering of miRNAs including additional intermediate risk group with selected blastemal components. (TIF) [file pone.0053417.s001.tif]
